# The Tegument of the Human Parasitic Worm *Schistosoma mansoni* as an Excretory Organ: The Surface Aquaporin SmAQP Is a Lactate Transporter

**DOI:** 10.1371/journal.pone.0010451

**Published:** 2010-05-03

**Authors:** Zahra Faghiri, Simone M. R. Camargo, Katja Huggel, Ian C. Forster, David Ndegwa, François Verrey, Patrick J. Skelly

**Affiliations:** 1 Molecular Helminthology Laboratory, Division of Infectious Diseases, Department of Biomedical Sciences, Cummings School of Veterinary Medicine, Tufts University, Grafton, Massachusetts, United States of America; 2 Institute of Physiology, University of Zurich, Zurich, Switzerland; University of Missouri-Kansas City, United States of America

## Abstract

Adult schistosomes are intravascular parasites that metabolize imported glucose largely via glycolysis. How the parasites get rid of the large amounts of lactic acid this generates is unknown at the molecular level. Here, we report that worms whose aquaporin gene (SmAQP) has been suppressed using RNAi fail to rapidly acidify their culture medium and excrete less lactate compared to controls. Functional expression of SmAQP in *Xenopus* oocytes demonstrates that this protein can transport lactate following Michaelis-Menten kinetics with low apparent affinity (Km = 41±5. 8 mM) and with a low energy of activation (*E_a_* = 7.18±0.7 kcal/mol). Phloretin, a known inhibitor of lactate release from schistosomes, also inhibits lactate movement in SmAQP-expressing oocytes. In keeping with the substrate promiscuity of other aquaporins, SmAQP is shown here to be also capable of transporting water, mannitol, fructose and alanine but not glucose. Using immunofluorescent and immuno-EM, we confirm that SmAQP is localized in the tegument of adult worms. These findings extend the proposed functions of the schistosome tegument beyond its known capacity as an organ of nutrient uptake to include a role in metabolic waste excretion.

## Introduction

Schistosomes are parasitic trematodes that infect over 200 million people worldwide and can cause the debilitating disease schistosomiasis [1]. Larval schistosomes called cercariae emerge from freshwater snail intermediate hosts and these can infect the final mammalian host via skin penetration. Parasites that move from a freshwater environment into the body of the mammalian host undergo a complex set of adaptive morphological and biochemical changes that is collectively called cercarial transformation. Cercariae transform into larval forms called schistosomula which enter the bloodstream and mature to the adult life stage. During transformation, the cercarial outer membrane is cast off and a new intravascular, double lipid bilayer covering is formed [2], [3]. The parasites also undergo a change in energy metabolism: free swimming cercariae metabolize glycogen stores via oxidative phosphorylation to CO_2_ and H_2_O whereas intravascular life forms metabolize glycogen largely via glycolysis, with the generation of lactic acid as the major end product [4], [5].

Adult schistosomes reside in mammalian mesenteric blood vessels. Here they import sugar from the bloodstream directly across their tegument and into their internal tissues using a number of membrane-spanning glucose transporter proteins [6], [7]. The adults consume copious quantities of glucose amounting to their dry weight every 5 hours [8]. Glucose catabolism via glycolysis leads to the generation of considerable amounts of lactic acid in schistosomes [9] which must be transported out of the cell to avoid poisoning metabolic pathways. Accumulation of lactic acid leads to a decrease in intracellular pH and cessation of glycolysis. Therefore, lactic acid must be efficiently eliminated if high rates of glycolysis are to be maintained. It has been estimated that as much as 0.043 mmol lactate/mg dry weight/hour is released by adult parasites cultured in medium containing 10 mM glucose [9]. The molecular mechanisms by which schistosomes rid themselves of this lactic acid are not known. In some systems, lactic acid transport is carried out by a family of proton-linked monocarboxylate transporters (MCTs) located at the plasma membrane [10], [11], [12]. Several MCT and MCT-related genes have so far been identified in mammals, each having a different tissue distribution [11], [12]. MCT genes have also been identified in other organisms of diverse phylogeny including *Saccharomyces cerevisiae*, *Plasmodium falciparum*, *Drosophila melanogaster* and *Caenorhabditis elegans*. We have examined the literature for reports of the presence of MCT homologs expressed in the tegumental membranes of intravascular schistosomes, through which lactic acid might be exported from the worms, and we failed to identify such molecules [13].

In our ongoing characterization of the schistosome host-interactive tegument, we cloned and characterized a cDNA encoding an aquaporin protein which we designated SmAQP [14]. Proteomic analysis of isolated tegumental membranes had earlier shown that the protein was localized there [15], [16]. SmAQP is a 304 amino acid membrane protein that is most highly expressed in the intravascular life stages (GenBank accession number EU780065). RNA interference (RNAi) experiments in which SmAQP gene expression was suppressed in the schistosomula life stage revealed that the protein was important for the control of water movement into and out of the parasites [14]. SmAQP-suppressed schistosomula exhibited lower viability in culture relative to control parasites and SmAQP-suppressed schistosomes had a generally more stunted appearance [14].

In this work, we examine the function of SmAQP in adult schistosomes. We confirm by immunolocalization, at the light and electron microscope level, that the protein is highly expressed in the adult tegument. Using RNAi with the adult parasites, and following the heterologous expression of SmAQP in *Xenopus* oocytes, we provide evidence that this protein can act as a conduit for lactate. These data extend the proposed functions of the tegument from that of its well characterized role in nutrient uptake to that of waste metabolite excretion. In addition this work provides a molecular explanation for the ability of intravascular schistosomes to eliminate an important and potentially toxic glycolytic end-product.

## Methods

### Parasites and mice

The Puerto Rican strain of *Schistosoma mansoni* was maintained at the Biomedical Research Institute, Rockville, MD, and obtained from Dr. Fred Lewis. Cercariae were obtained from infected *Biomphalaria glabrata* and used to infect Balb/c mice at ∼125 cercariae/mouse [17]. Adult male and female parasites were recovered by perfusion from the mice 7 weeks later. Parasites were cultured in complete RPMI culture medium which is RPMI medium supplemented with 10 mM Hepes, 2 mM glutamate, 5% fetal calf serum and antibiotics (100 U/ml penicillin and 100 µg/ml streptomycin) at 37°C, in an atmosphere of 5% CO_2_.

### Preparation and delivery of dsRNA

Synthetic small inhibitory RNAs (siRNAs) were used, as described previously [14]; one siRNA targets the SmAQP gene and a negative control siRNA targets no sequence in the *S. mansoni* genome. Adult parasites (10/group) in 100 µl electroporation buffer (Ambion, TX), received siRNAs (5 µg) by electroporation in a 4 mm cuvette by applying a square wave with a single 20 ms impulse, at 125 V and at room temperature, as described [18]. Parasites were then transferred to 1 ml complete RPMI. Culture medium was replaced every 2 days. Gene suppression was confirmed by quantitative real-time PCR (qRT-PCR) and changes in protein level were examined by western blot analysis and immunofluorescence microscopy, at least 7 days after treatment.

### Gene expression analysis

To monitor the expression of the SmAQP gene following siRNA treatment, RNA was first extracted from the parasites using the Trizol method (Invitrogen, CA) following the manufacturer's instructions. cDNA was synthesized using 1 µg RNA and an oligo (dT)_20_ primer and Superscript reverse transcriptase (Invitrogen, CA). Quantitative real time PCR was performed using custom TaqMan Assays with the primer sets and reporter probes labeled with 6-carboxyfluorescein (FAM), obtained from Applied Biosystems (Foster City, CA) and assay conditions, as described previously [14]. For relative quantification, the ΔΔCt method was employed, using alpha tubulin as the endogenous standard for each sample. Results obtained from parasites treated with irrelevant siRNA were used for calibration [19]. For graphical representation, the ΔΔCt values were normalized to controls and expressed as percent difference [19].

### Western blot analysis

Adult parasite lysates were prepared by adding 50 µl of ice cold, cell disruption buffer (PARIS Kit, Ambion, TX) followed by incubation for 30 min on ice. The protein content in each extract was estimated using the BCA Protein Assay Kit (Pierce, IL) according to the manufacturer's instructions. Five µg of each extract was resolved on NuPAGE 4–12% Bis-Tris ready-made gels (Invitrogen, CA). Protein transfer to polyvinylidene fluoride membrane and detection of SmAQP using the affinity purified anti-SmAQP antiserum was achieved by standard means, as described [14].

### Protein immunolocalization

Immunofluorescent detection of SmAQP in acetone-fixed whole parasites, or in 7 µm acetone-fixed, adult parasite frozen sections, was carried out using an affinity-purified, anti-SmAQP antiserum, essentially as described [14]. The serum was raised in rabbits that had been injected with a synthetic peptide derived from the carboxyl terminus of SmAQP [14]. Alexa fluor 488-conjugated goat anti-rabbit IgG (Invitrogen) was used as secondary antibody, as in earlier work [20].

### Immunogold Labeling and Electron Microscopy

Freshly perfused adult parasites were fixed overnight with 2% glutaraldehyde in 0.1 M cacodylate buffer at 4°C. The samples were then dehydrated in a graded series of ethanol, then infiltrated and embedded in L.R. White acrylic resin. Ultramicrotomy was performed using a Leica Ultracut R ultramicrotome and the sections collected on gold grids. Grids were immunolabeled in a two step method according to the following procedure; the grids were conditioned in phosphate buffered saline (PBS) for 5 min ×3 at room temperature (RT), followed by the blocking of non-specific labeling for 30 min at RT using 5% non-fat dry milk in PBS. After rinsing, the grids were exposed to anti-SmAQP antibodies diluted 1∶30 for 1 hour at RT, followed by washing in PBS and then incubated with the secondary antibodies diluted 1∶30 (10 nm gold-labeled goat anti-rabbit IgG (H&L, Amersham Biosciences)) for 1 hour at RT, and finally rinsed thoroughly in water. The grids were exposed to osmium vapor and/or lightly stained with lead citrate to improve contrast and were examined and photographed using a Philips CM 10 electron microscope at 80 KV.

### pH measurement and lactate assay

Ten adult schistosomes were treated with SmAQP siRNA or control siRNA or no siRNA as described above and cultured in 1 ml complete RPMI medium. Forty eight hours later the medium was replaced. Forty eight hours later the color of the medium in which the SmAQP siRNA-treated parasites were cultured was observed to be noticeably different compared with the color of the medium from both control groups, suggesting a more alkaline environment. To test this notion, the pH of the media from triplicate wells containing control and SmAQP siRNA-treated parasites was measured using an Oakton Acorn pH Meter (Vermon Hill, IL, USA). In addition, lactate levels in the media were measured directly using a Lactate Assay Kit following the manufacturer's instruction (Eton Bioscience Inc., San Diego, CA). Briefly, media were diluted 1∶50 with distilled water and 20 µl was added to 50 µl lactate assay solution in a 96-well flat bottom microplate. This was incubated for 30 min at 37°C. The reaction was stopped by adding 50 µl 0.5 M acetic acid and the absorbance at 490 nm was measured on a Synergy HT Multi-Mode Microplate Reader (Winooski, VT). The lactate concentration in each well was determined relative to a standard curve generated from a lactate standard sample supplied with the kit.

### Functional analysis of SmAQP expressed in *Xenopus laevis* oocytes

In order to express SmAQP in *Xenopus* oocytes, the entire SmAQP coding DNA was first amplified by PCR using adult parasite cDNA and oligonucleotides, SmAqp-XO1 (5′-CGCCTCGAGATGGTTTCGTGTTCTGAAAAATATGC-3′) and SmAqp -XO2 (5′-GCGGGATCCTTACGGTGATGAATAGGCCACCAAC-3′), using conditions as described [6]. Underlined sequences denote restriction sites; XhoI for SmAqp-XO1 and Bam HI for SmAqp-XO2. Next, the PCR product was gel purified, digested with XhoI and Bam HI and ligated into the similarly digested *Xenopus* expression plasmid, pSDeasy. TOP10 *E. coli* cells (Invitrogen Inc., CA) were transformed with the ligation mixture and recombinant transformants were selected on agar plates containing 100 µg/ml ampicillin. Plasmid was purified from several clones and the cloned inserts were sequenced. One plasmid, pNBA001, was linearized by digestion with PstI and cRNA was synthesized *in vitro*, as described earlier [21]. *Xenopus* oocytes were injected with 25 ng of SmAQP cRNA, and non-injected (NI) oocytes were used as negative controls. Oocytes were then incubated at 18°C for 1–2 days in Barth's solution and radiotracer studies were performed essentially as described [22]. Label uptake per injected oocyte was measured 1.5 minutes after substrate addition. The values obtained for the non-injected oocytes were subtracted from SmAQP expressing oocytes. For the selectivity experiments, 1 mM of solute (mannitol, lactate, glucose, fructose, L-alanine) was used in the presence of the respective radiolabeled substance (20 µCi/ml ^3^H-glucose, fructose and L-alanine, or 2 µCi/ml mannitol and lactate (Hartmann, Braunschweig, Germany)) at 25°C. The pH dependency of lactate transport was assayed at pH 7.4 and 6.3, in the presence of 1 mM lactate. For the lactate dose dependence experiments, 100, 33, 10, 1 and 0.1 mM were used.

The activation energy (*E_a_*) was calculated by plotting the temperature dependent transport (log of the values) as an Arrhenius plot and determining the slope of the curve, as previously described [23]. With the logarithmic form of the Arrhenius equation and linear regression analysis applied to experimental data, *E_a_* can be calculated as: *- E_a_ = K×2.303×R*, where R is the gas constant (1.9858775 kcal K^−1^ × mol^−1^) and the slope (K) is calculated from the Arrhenius plot. The experiments were performed at 4, 15 and 25°C in the presence of 1 mM lactate. The coefficient of permeability diffusion (*Ps*) was calculated as described [24] from the formula: *Ps = N/(A×Δc)*, where *N* is the radiolabeled transport of lactate (pmol/s); *A* is the average geometric area of an oocyte (0.045 cm^2^), and *Δc* is the concentration of the solute in pmol/cm^3^. P*s* is expressed in 10^−6^ cm/s. The water osmotic permeability (*P_f_)* was calculated as previously described [25]. Briefly, the oocytes were transferred from an isotonic (206 mOsm/Kg) to a hypotonic solution (72 mOsm/Kg). In some experiments oocytes were first incubated for 10 minutes in isotonic solution containing phloretin (0.5 mM), a compound that has been shown to inhibit aquaporin function [24]. After this period they were transferred to hypotonic solution with the same concentration of phloretin. The projected surface area was recorded for 1 minute every 1.5 seconds by videomicroscopy. The area from each 5 seconds were plotted and fitted to a quadratic polynomial equation, and the initial swelling rates were calculated (*d(V/V_0_)/dt* in s^−1^). The water osmotic permeability was calculated from the initial swelling rate (*d(V/V_0_)/dt*), the surface area of the oocyte (*S* = 0.045 cm^2^), the molar ratio of water (*V_w_* = 18 cm^3^/mol) and osmotic gradient (*osm_in_−osm_out_*) using the following formula: *P_f_ = [V_0_×d (V/V_0_)/dt]/[S×V_w_×(osm_in_−osm_out_)]*.

The results are expressed in 10^−4^ cm/s.

In some experiments, lactate transport was assayed in the presence of 0.5 mM phloretin or 0.3 mM HgCl_2_. The dose dependence inhibition curve was assayed with 0.5, 0.05, 0.005 and 0.0005 mM phloretin. Lactate was used at 1 mM and experiments performed at 25°C. The data were fitted by an inhibition curve with variable slope and the K*_i_* calculated.

### Statistical Analysis

The Student's t-test was used to determine the statistical significance of differences between groups.

## Results

**Figure 1:**
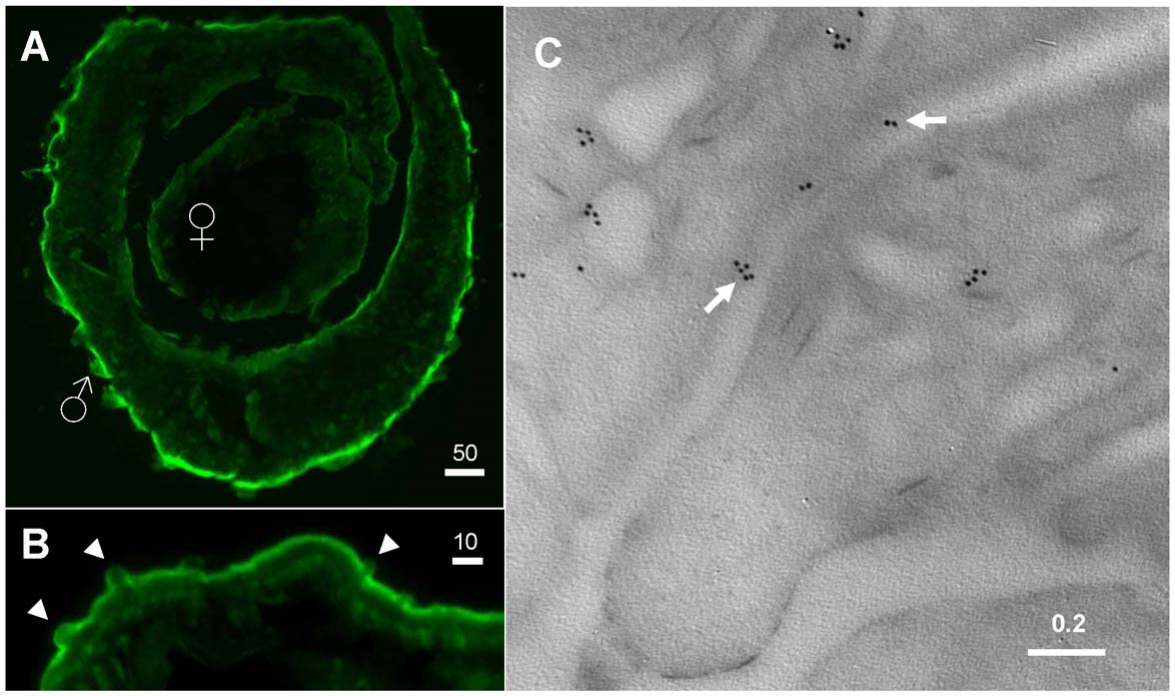
Immunolocalization of SmAQP in adult parasites. A. Cross section through a male/female couple showing strong staining with anti-SmAQP antibodies in the tegument. B. Higher magnification image of the peripheral tissue of an adult male showing diminished staining in the tegumental tubercles (arrowheads). C. Electron micrograph of the adult tegument showing immunogold labeling of SmAQP. Arrows indicate gold particles at the host/parasite interface. Numbers above scale bars represent microns.

Figure 1 shows the immunolocalization pattern of SmAQP in a section of an adult schistosome pair. It is clear that the protein is prominently expressed in the tegument of males and females. Most intense staining is seen in the dorsal surface of the male. Figure 1B shows a higher magnification image of this area. Less intense staining is seen in the tegumental tubercles (arrowheads, figure 1B). Immuno-electron microscopy confirms the distribution of SmAQP within the tegument, including at the host-parasite surface (arrows, figure 1C). The immunogold particles are frequently clustered.

**Figure 2:**
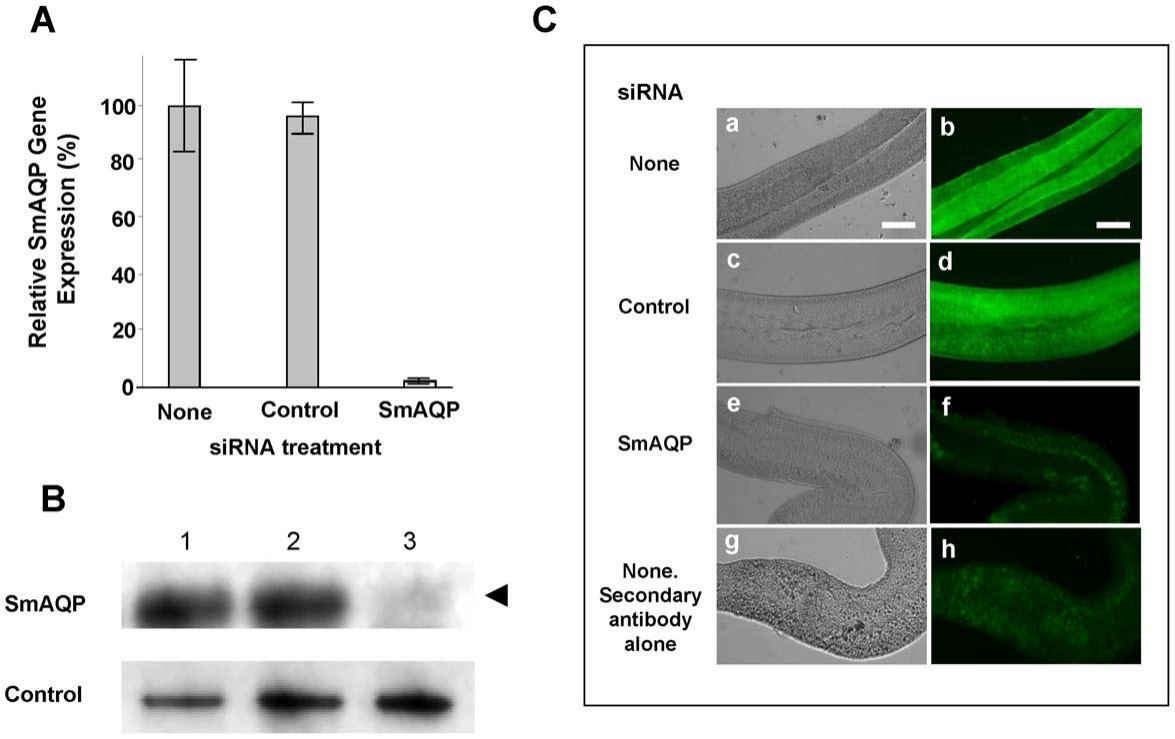
Changes in SmAQP expression. A. Relative SmAQP expression (mean ± SE) in adult schistosomes 7 days after treatment with none, or control, or SmAQP siRNA. B, Detection by western analysis of SmAQP protein (top panel), and a control protein (SPRM1hc, bottom panel) in extracts prepared from parasites 28 days after treatment with none (lane 1), control (lane 2), or SmAQP (lane 3) siRNA. The arrowhead indicates the diminished level of SmAQP protein seen in lane 3. C. Localization of SmAQP in whole, fixed adult parasites stained with anti-SmAQP antiserum (a–h) 14 days after no (a,b), control (c,d), or SmAQP (e,f) siRNA treatment. An additional control group (g,h) was untreated with siRNA and stained with secondary antibody alone. Panels a,c,e,g are phase contrast images while panels b,d,f,h illustrate antibody binding. Non suppressed parasites (panels a–d) stain brightly for SmAQP while the SmAQP-suppressed parasites (e,f) and parasites stained with secondary antibody alone (g,h) stain very weakly. The bar represents 100 µm.

SmAQP gene expression was suppressed in adult parasites *in vitro* by introducing a target specific siRNA using electroporation. Figure 2A shows the specific and robust suppression of SmAQP (95%), measured by qRT-PCR, 7 days after treatment. SmAQP gene suppression levels of >90% are maintained in cultured parasites for at least 28 days (not shown). Western blot analysis demonstrates that this treatment also results in substantial diminution in SmAQP protein production (figure 2B). Lower levels of SmAQP protein are detected (arrow, figure 2B) in extracts from SmAQP siRNA-treated parasites versus controls, 28 days post treatment. As illustrated in figure 2C, immunostaining whole fixed parasites using anti-SmAQP antiserum further confirms the robust knockdown of SmAQP protein. Control parasites (either untreated or treated with in irrelevant siRNA shown in figure 2C panels a–d) reveal strong staining with anti-SmAQP antibody whereas parasites treated with SmAQP siRNA stain very poorly (figure 2C, panel e,f), and are more similar to untreated parasites, incubated with secondary antibody alone (figure 2C, panel g,h). These parasites were stained 14 days after RNAi treatment.

**Figure 3:**
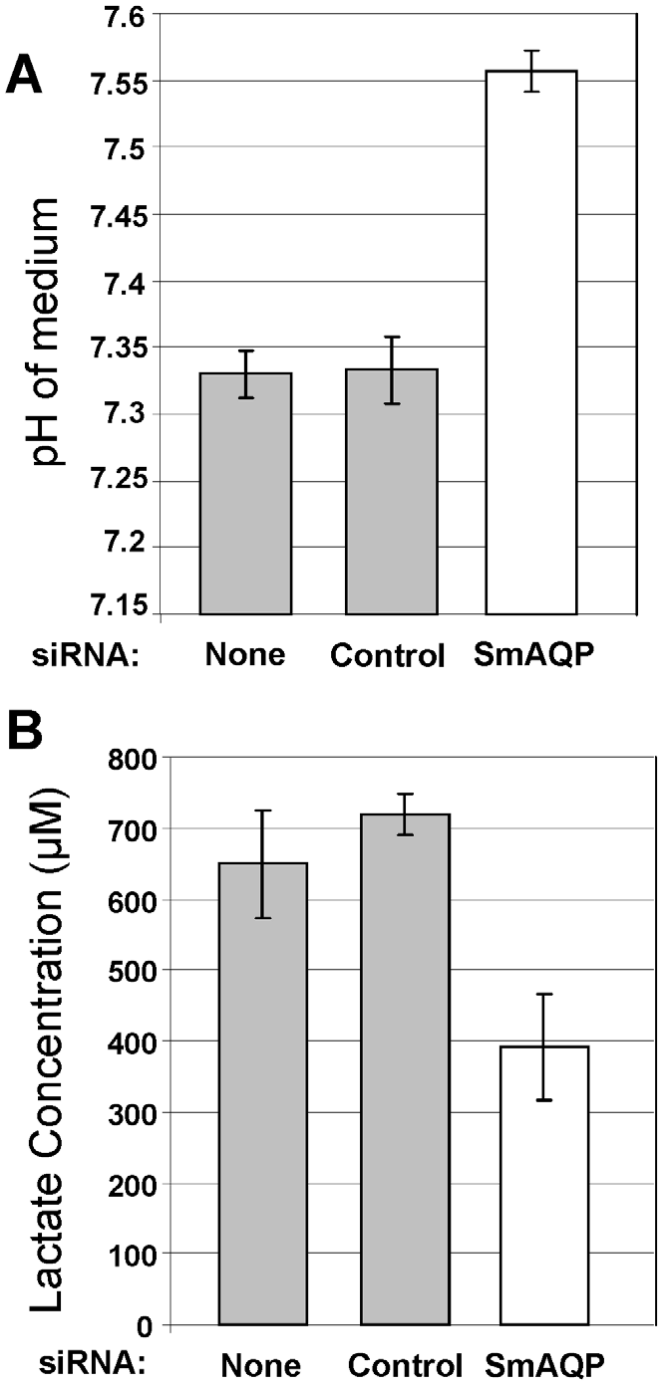
Changes in the medium of SmAQP suppressed and control adult schistosomes. A. pH (mean ± SE) of 1 ml complete RPMI medium containing 10 adult parasites that were treated with none or control or SmAQP siRNA 96 hours earlier. B. Lactate concentration (µM) in medium containing 10 adult parasites that were treated with none or control or SmAQP siRNA 96 hours earlier.

This robust suppression of SmAQP does not result in any detectable change in adult parasite morphology or behavior. However, adult parasites whose SmAQP expression is suppressed by RNAi treatment, unlike controls, fail to rapidly acidify their culture medium. Figure 3A shows the pH measurements of media that contained SmAQP-suppressed versus control parasites after 96 hours in culture. It is clear that the medium containing control parasites is significantly more acidic (grey bars, figure 3A) compared to the medium containing the SmAQP-suppressed worms (white bar, figure 3A, p<0.001). Lactic acid is mostly dissociated to lactate^−^ and H^+^ at normal near-neutral, intracellular pH, and figure 3B shows that the medium from the SmAQP-suppressed group (figure 3b, white bar) has a significantly lower lactate content compared with either control group (figure 3B, grey bars, p<0.001). This result shows that SmAQP suppression impairs lactate elimination

**Figure 4:**
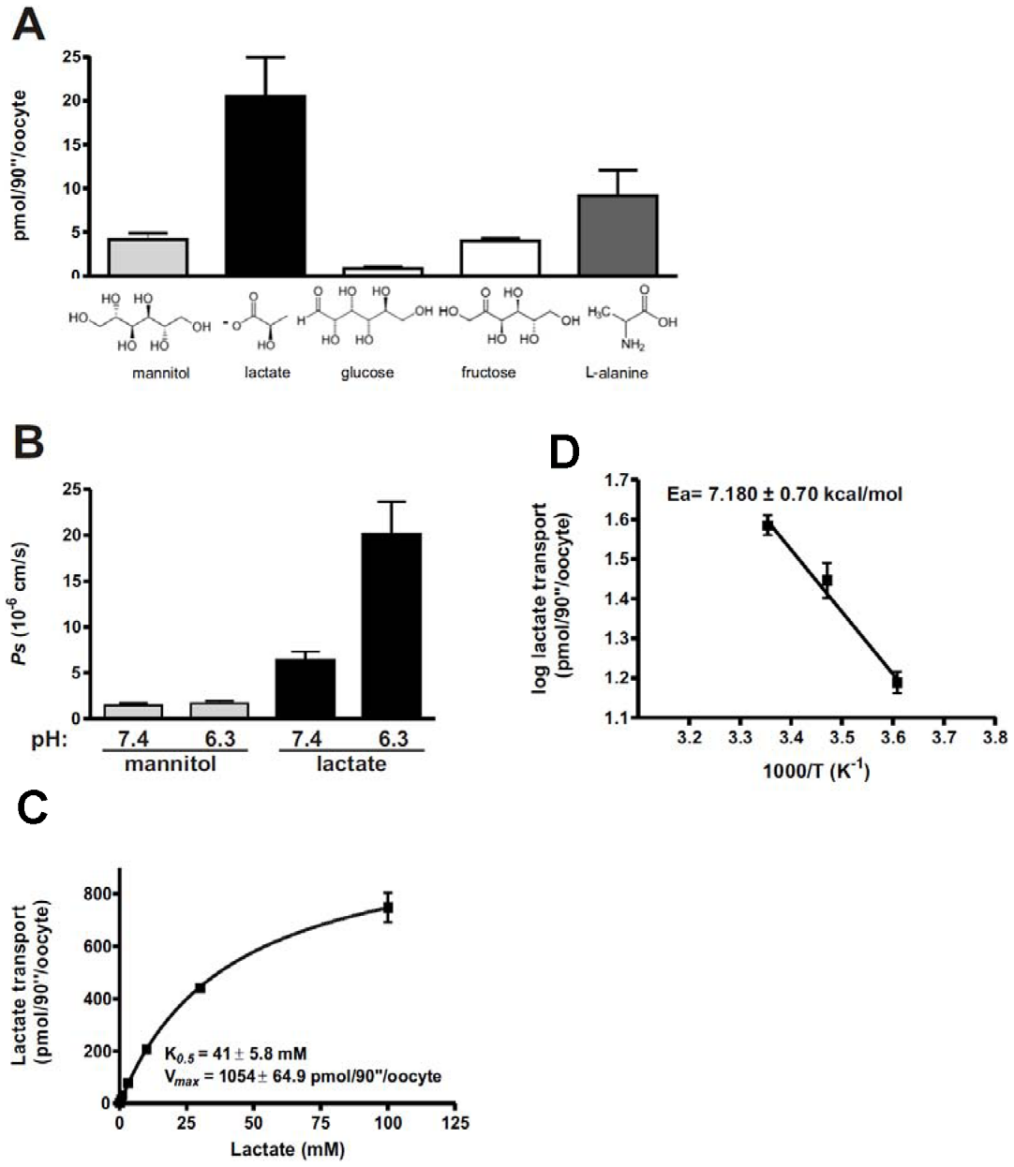
SmAQP is an aquaglyceroporin. A. SmAQP, when expressed in *X. laevis* oocytes, can transport several solutes. SmAQP transports lactate, in addition to mannitol, fructose and L-alanine but not glucose. The substrates were added to a final concentration of 1 mM and accumulation of radiolabeled substance traces on the cells was measured after 1.5 minute (n = 16–18 oocytes). B. The transport of lactate is pH dependent. Solute diffusion coefficient permeability for lactate increased in a pH dependent manner (6.4±0.9×10^−6^ cm/s pH 7.4 and 20.1±3.5×10^−6^ cm/s pH 6.3), whereas for mannitol it was unchanged (1.5±0.2×10^−6^ cm/s pH/.4 and 1.7±0.2×10^−6^ cm/s pH 6.3) (n = 15–17). C. The transport of lactate is saturable. SmAQP has low affinity for lactate and follows Michaelis-Menten kinetics (n = 14–24). D. Lactate moves through a pore. The energy of activation (*Ea*) was estimated by plotting the temperature dependent transport (at 4, 15 and 25°C) as an Arrhenius plot. The slope of the linear regression was −1.57±0.15 K (n = 9–15). In all cases, values obtained from control oocytes, not-expressing SmAQP have been subtracted to generate the data shown. Data represent the mean ± SE generated in at least 2 independent experiments.

To directly test the hypothesis that SmAQP can transport lactate, the protein was expressed in *Xenopus* oocytes and the uptake of radiolabeled lactate was compared in SmAQP-expressing versus control oocytes. Figure 4 shows the substrate specificity of SmAQP. The highest transport rate was observed for lactate, but other molecules - mannitol, fructose and the amino acid L-alanine, were also transported (figure 4A). Of the substrates tested, it is notable that the 3-carbon substrates are transported in greater amounts compared to the 6-carbon substrates. Glucose was not transported in this system. Lactate, but not mannitol, permeability (*Ps*), was increased 3 fold at pH 6.3 when compared with pH 7.4 (figure 4B), suggesting the protonated form is transported. The transport of lactate follows Michaelis-Menten kinetics with very low apparent affinity (Km = 41±5. 8 mM) (figure 4C). Lactate crosses the membrane through a pore, as it is evident by the low energy of activation (*E_a_* = 7.18±0.7 kcal/mol) estimated from the Arrhenius plots (figure 4D).

**Figure 5:**
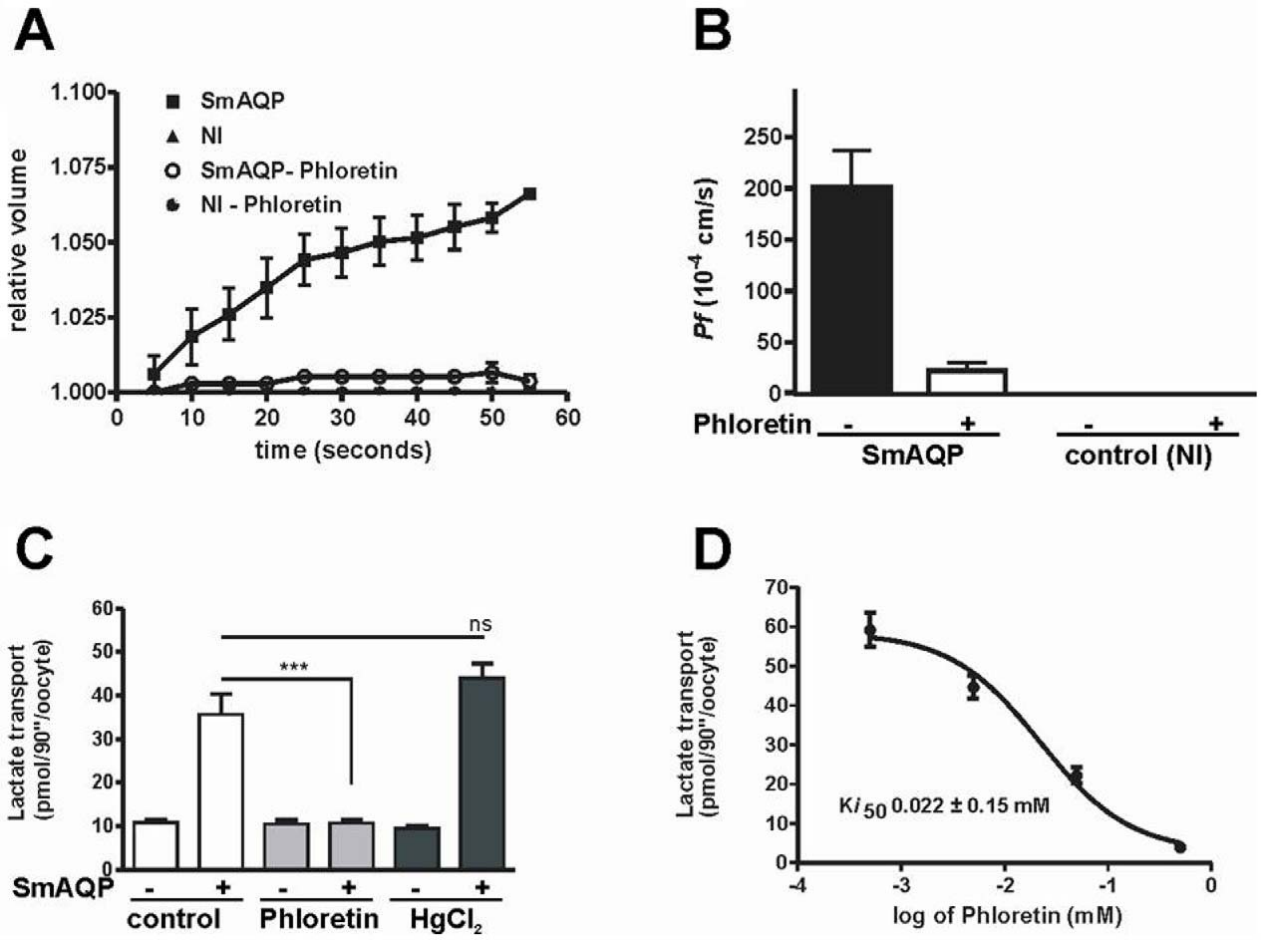
A–B. Increase in water osmotic permeability in oocytes expressing SmAQP. A. Volume increase in control, non- injected (NI) oocytes versus oocytes expressing SmAQP when transferred from normal salt solution (206 mOsm/Kg) to a hypotonic solution (72 mOsm/Kg) in the presence or absence of phloretin (0.5 mM). B. The osmotic permeability coefficient *Pf* of oocytes expressing SmAQP or control, non-injected (NI) oocytes in the presence (+) or absence (−) of phloretin (0.5 mM) (n = 3–6). C–D. Phloretin inhibition of water permeability and solute diffusion. C. The transport of lactate is inhibited by phloretin (0.5 mM, grey bars), but not by HgCl_2_ (0.3 mM, black bars). “+” signifies oocytes expressing SmAQP, “−” indicates control, non-injected oocytes. D. Kinetics of phloretin inhibition of lactate transport. Phloretin inhibits lactate movement with high affinity (K*i*50 = 22 µM, n = 15–16). Values obtained from control oocytes, not-expressing SmAQP have been subtracted to generate the data shown. Data represent the mean ± SE generated in at least 2 independent experiments.

SmAQP is also permeable to water; the oocytes expressing SmAQP, when transferred from an isotonic to a hypotonic solution, swelled and an increase in volume (surface area) was observed during the first minute as depicted in figure 5A. The water osmotic permeability (*P_f_*) in oocytes expressing SmAQP was 202±35.65×10^−4^ cm/s (figure 5B). Oocytes expressing SmAQP burst in less than 3 minutes when incubated in hypotonic solution; non-injected oocytes survived at least until the last measurement at 90 minutes (data not shown). Oocyte swelling in water was inhibited by phloretin (figures 5A–B), and the water osmotic permeability coefficient in oocytes expressing SmAQP was inhibited to almost 90%. The non-injected (NI) oocytes or non-injected oocytes treated with phloretin showed no appreciable alteration in volume (figure 5A–B). Lactate transport into SmAQP-expressing oocytes, as for water transport, could be 100% inhibited by phloretin, as depicted in figures 5C–D. The half maximal inhibitory concentration (K*_i_*) of phloretin is 22 µM (figure 5C). Mercury chloride did not inhibit lactate transport but rather increased it (figure 5C). This increase is not statistically significant.

## Discussion

Intravascular schistosomes import and consume copious quantities of glucose across their teguments [8], [26]. Despite the fact that the adults possess functional mitochondria and are capable of oxidative phosphorylation, most glucose catabolism is via glycolysis and the adults have long been termed homolactate fermenters [26], [27], [28]. The molecular mechanisms by which adult schistosomes eliminate the substantial quantities of lactate they generate via glycolysis have not been described [9], [10]. Members of the monocarboxylate transporter protein (MCT) family have been shown capable of transporting lactate in other systems [10], [11], [12]. However, no homolog belonging to the MCT protein family has been identified in any analysis of the schistosome tegument through which the parasites might export lactate [15].

In our analysis of SmAQP function in schistosomes, we made the serendipitous discovery that adult parasites whose SmAQP expression is suppressed by RNAi treatment, unlike controls, fail to rapidly acidify their culture medium; there is minimal change in the phenol red indicator in RPMI medium after 96 h in culture. Therefore the medium that contained SmAQP-suppressed worms simply looked redder than media containing either of the control groups of worms. Direct measurement of the pH of the media confirmed that media containing control worms was significantly more acidic than medium that contained the SmAQP-suppressed group. Since one major reason cultured schistosomes rapidly acidify their medium is by the excretion of lactic acid [9], we hypothesized that the SmAQP-suppressed parasites were deficient in this capability. To test this, the level of lactate in parasite culture medium was measured and, as predicted, medium from the SmAQP-suppressed group was found to contain significantly less lactate compared to media from control parasites.

SmAQP in schistosomula is involved in osmoregulation and volume control [14]. Suppressing SmAQP gene expression debilitates schistosomula; significantly fewer SmAQP-suppressed parasites survive in culture (beyond 14 days) and those that do tend to have a stunted appearance [14]. We do not observe any similar diminution in adult schistosome viability following SmAQP-suppression even after prolonged (28 days) maintenance in culture, nor do we detect gross morphological or behavioral differences between SmAQP-suppressed versus control worms. However, SmAQP-suppression in adult schistosomes leads to an extended phenotype that is evident in the culture medium, as documented above. Given the known role of SmAQP in such a fundamental process as water movement, the phenotype measured for the SmAQP-suppressed worms (less acid medium, less lactate in the medium) could be an indirect consequence of interfering in the crucial process of water distribution. To directly test the hypothesis that SmAQP can transport lactate, the protein was expressed in *Xenopus* oocytes and lactate uptake assays were performed. As reported, SmAQP-expressing oocytes import significantly more lactate versus controls. These data unequivocally demonstrate the ability of SmAQP to transport this substrate.

Members of the aquaporin (AQP) family have traditionally been divided into two classes, the AQPs, pure water channels that only conduct water (e.g. human proteins AQP1,2,4,5,6,8), and the aquaglyceroporins, channels that also conduct small solutes, notably glycerol (e.g. human proteins AQP3,7,9,10) [24], [29], [30]. Sequence comparison of SmAQP shows that it belongs to the aquaglyceroporin family [14] and this suggests that its permeability extends beyond water alone. The work reported here shows that SmAQP is indeed an aquaglyceroporin, permeable to water and solute. SmAQP was permeable not only to water and lactate but to mannitol Interestingly SmAQP was also permeable to sugar like fructose and the small neutral amino acid, L-alanine. SmAQP is not permeable to glucose which we have shown before is transported by specific facilitated diffusion glucose transporter proteins (SGTPs) [6]. SmAQP showed the highest permeability to lactate, and in particular to its protonized form. The permeability increased 3 fold with the lowering of the pH and so increasing the concentration of the protonized form from 0.3 and 3.6 µM in 1 mM lactate solution at pH 7.4 and 6.3 respectively. Lactate and its protonized form were similarly shown to be transported by the mammalian aquaglyceroporin AQP9 [24]. The transport of lactate in SmAQP-expressing oocytes follows Michaelis-Menten kinetics; it is saturable, as is AQP9 [31]. The presence of SmAQP in the tegument of the worms assures an efficient efflux of lactate from the animals to the host-medium. Worms in culture can excrete in the medium 0.5 mmol per hour of lactate and they can accumulate in their bodies a high concentration of lactate [8], [10]. It is noteworthy that the low affinity of SmAQP for lactate (Km = 41 mM) is similar to that of the rat lactate transporter MCT4, expressed in *Xenopus* oocytes, whose Km = 34 mM [32]. The MCT4 protein is reported to be adapted to the export of lactate from highly glycolytic cells [32]. As already mentioned, adult schistosomes are also highly glycolytic.

The energy of activation (*Ea*) estimated from Arrhenius plots for lactate (7.18±0.70 kcal/mol in *X. laevis* oocytes) is consistent with the values found for mammalian aquaglyceroporin orthologs (5.3–12 kcal/mol) and provide support for the notion that lactate crosses membranes through a pore [24], [33], [34]. As shown in our previous work [35], SmAQP is responsible for the osmotic swelling of the worms in hypotonic solution. Using the *X. laevis* oocytes as a heterologous system for express SmAQP, we show in this work that SmAQP also increases the water osmotic permeability in cells expressing the parasite protein. Additionally, we observe that the swelling of the oocytes can be inhibited by phloretin (figure 5C). Phloretin effectively reduced the osmotic permeability (90%) as well as the transport of lactate (100% at 0.5 mM). Phloretin has previously been reported to inhibit lactate release from cultured adult worms [10]. The similar inhibition profiles of solute and water permeabilites in SmAQP-expressing oocytes suggest that solute and water share a single pore, as already observed for the mammalian aquaglyceroporins, AQP9 [24].

Some mammalian aquaglyceroporins and aquaporins can be inhibited by mercury chloride (HgCl_2_). For instance, HgCl_2_ reduced the water permeability of the mammalian proteins AQP1, AQP2, AQP3 and AQP9 [24], [36], [37] but not the aquaglyceroporin AQP7 [34]. Additionally, HgCl_2_ reduced the transport of glycerol but not of urea in oocytes expressing rat AQP9 [24]. In our studies, lactate transport in oocytes expressing SmAQP was not reduced in the presence of HgCl_2_. Changes in the water permeability (relative volume) of oocytes and schistosomes in hypotonic solution were also not inhibited by the presence of HgCl_2_ (data not shown). The insensitivity of SmAQP solute and water permeability to HgCl_2_ may be explained by the fact that the SmAQP protein has an L-alanine and not an L-cysteine residue at position 219. This residue is localized only 3 amino acid residues before the second conserved “NPA” motif which forms the pore of the channel, and has been suggested to be responsible for HgCl_2_ binding and inhibition of some aquaporins [35], [38].

The work reported here shows clearly that lactate is among the substrates SmAQP can transport. Demonstrating the transport versatility of SmAQP, in addition we show that mannitol and fructose and alanine can also enter cells via this pore. We have previously provided evidence that SmAQP can transport the drug potassium antimonyl tartrate (PAT) [35]. As is the case for other aquaporins, it is possible that SmAQP can act as a conduit for yet other, additional metabolites.

While members of both the aquaporin and monocarboxylate transporter (MCT) families can transport lactate, the two protein families are quite distinct in terms of their primary sequences and predicted conformations. Aquaporin proteins contain six membrane-spanning domains and while each protein can form an independent pore, every AQP studied to date forms a tetramer [29]. Of the AQPs studied, only one, AQP9 (in addition to SmAQP) has been shown to transport lactate [24]. In contrast, several members of the MCT family have been shown capable of transporting lactate [11]. MCT proteins differ from aquaporins by being typically larger and possessing 12 membrane-spanning domains. The substrate specificities of different members of the two protein families can be very divergent and, with the exception of lactate, are generally reported not to overlap [11], [29].

The wide distribution of SmAQP throughout the tegument of adult schistosomes suggests that lactate is eliminated via SmAQP directly across the tegument of schistosomes and into the host bloodstream. This provides a wide area for the disposal of this metabolite. The relatively high expression of SmAQP at the male dorsal surface may be the result of a preference to eliminate this toxic glycolytic product into the blood stream of the host via this surface and not predominantly via the ventral surface into the gynaecophoric canal–a space often occupied by the male's female partner.

Following immuno-electron microscopy we detect SmAQP by immunogold labeling and observe gold particles often clustered in the parasite tegument. This is consistent with the notion of SmAQP protein clustering and the presence of zones within the tegument of metabolite movement. The data reported here provide evidence that the function of the tegument extends from its well characterized role in nutrient uptake to include waste metabolite excretion. This raises questions concerning the precise role of the presumed excretory system of platyhelminths–the protonephridial system.

In summary, this work shows that tegumental SmAQP can transport nutrients as well as a metabolic waste product. The work suggests that the substantial lactate generated by glycolysis in blood-dwelling schistosomes can be excreted across the parasite tegument via the tegumental membrane protein SmAQP. These data provide a molecular understanding of how schistosomes cope with the large quantities of lactate generated from the largely anaerobic carbohydrate metabolism that is a hallmark of their intravascular lives [4], [5], [8], [21], [39].

## References

[1] Gryseels B, Polman K, Clerinx J, Kestens L (2006). Human schistosomiasis.. Lancet.

[2] Skelly PJ, Shoemaker CB (2000). Induction cues for tegument formation during the transformation of *Schistosoma mansoni* cercariae.. Int J Parasitol.

[3] Skelly PJ, Shoemaker CB (2001). The *Schistosoma mansoni* host-interactive tegument forms from vesicle eruptions of a cyton network.. Parasitology.

[4] Horemans AM, Tielens AG, van den Bergh SG (1991). The transition from an aerobic to an anaerobic energy metabolism in transforming *Schistosoma mansoni* cercariae occurs exclusively in the head.. Parasitology.

[5] Tielens AG, Verwijs A, Elfring RH, van den Heuvel JM, van den Bergh SG (1989). *Schistosoma mansoni*: rapid turnover of glycogen by adult worms in vivo.. Exp Parasitol.

[6] Skelly P, Cunningham J, Kim J, Shoemaker C (1994). Cloning, characterization and functional expression of cDNAs encoding glucose transporter proteins from the human parasite, *Schistosoma mansoni*.. J Biol Chem.

[7] Skelly PJ, Shoemaker CB (1996). Rapid appearance and asymmetric distribution of glucose transporter SGTP4 at the apical surface of intramammalian-stage *Schistosoma mansoni*.. Proc Natl Acad Sci U S A.

[8] Bueding E (1950). Carbohydrate metabolism of *Schistosoma mansoni*.. J Gen Physiol.

[9] Githui EK, Damian RT, Aman RA (2006). *In vitro* efflux of lactic acid by schistosomes cultured in varying concentrations of glucose: potential toxicity of accumulated lactic acid.. J Trop Microbiol Biotechnol.

[10] Githui EK, Damian RT, Aman RA (2006). *Schistosoma mansoni*: biochemical characterization of lactate transporters or similar proteins.. Exp Parasitol.

[11] Merezhinskaya N, Fishbein WN (2009). Monocarboxylate transporters: past, present, and future.. Histol Histopathol.

[12] Meredith D, Christian HC (2008). The SLC16 monocaboxylate transporter family.. Xenobiotica.

[13] Skelly P, Wilson R (2006). Making Sense of the Schistosome Surface.. Advances in Parasitology.

[14] Faghiri Z, Skelly PJ (2009). The role of tegumental aquaporin from the human parasitic worm, *Schistosoma mansoni*, in osmoregulation and drug uptake.. FASEB J.

[15] Braschi S, Curwen RS, Ashton PD, Verjovski-Almeida S, Wilson A (2006). The tegument surface membranes of the human blood parasite *Schistosoma mansoni*: a proteomic analysis after differential extraction.. Proteomics.

[16] Braschi S, Wilson RA (2006). Proteins exposed at the adult schistosome surface revealed by biotinylation.. Mol Cell Proteomics.

[17] Da'Dara AA, Skelly PJ, Walker CM, Harn DA (2003). A DNA-prime/protein-boost vaccination regimen enhances Th2 immune responses but not protection following *Schistosoma mansoni* infection.. Parasite Immunol.

[18] Correnti JM, Brindley PJ, Pearce EJ (2005). Long-term suppression of cathepsin B levels by RNA interference retards schistosome growth.. Mol Biochem Parasitol.

[19] Livak KJ, Schmittgen TD (2001). Analysis of relative gene expression data using real-time quantitative PCR and the 2(-Delta Delta C(T)) Method.. Methods.

[20] Skelly PJ, Dougan PM, Maule A, Day TA, Shoemaker CB (2001). Cloning and characterization of a muscle isoform of a Na,K-ATPase alpha subunit (SNaK1) from *Schistosoma mansoni*.. Parasitology.

[21] Mastroberardino L, Spindler B, Pfeiffer R, Skelly PJ, Loffing J (1998). Amino-acid transport by heterodimers of 4F2hc/CD98 and members of a permease family.. Nature.

[22] Krautz-Peterson G, Camargo S, Huggel K, Verrey F, Shoemaker CB (2007). Amino Acid Transport in Schistosomes: Characterization of the Permease Heavy Chain SPRM1hc.. J Biol Chem.

[23] Bacconi A, Ravera S, Virkki LV, Murer H, Forster IC (2007). Temperature dependence of steady-state and presteady-state kinetics of a type IIb Na+/P i cotransporter.. J Membr Biol.

[24] Tsukaguchi H, Shayakul C, Berger UV, Mackenzie B, Devidas S (1998). Molecular characterization of a broad selectivity neutral solute channel.. J Biol Chem.

[25] Zhang RB, Logee KA, Verkman AS (1990). Expression of mRNA coding for kidney and red cell water channels in *Xenopus* oocytes.. J Biol Chem.

[26] Skelly PJ, Tielens AGM, Shoemaker CB (1998). Glucose transport and metabolism in mammalian stage schistosomes.. Parasitol Today.

[27] Shapiro TA, Talalay P (1982). *Schistosoma mansoni*: mechanisms in regulation of glycolysis.. Exp Parasitol.

[28] Thompson DP, Morrison DD, Pax RA, Bennett JL (1984). Changes in glucose metabolism and cyanide sensitivity in *Schistosoma mansoni* during development.. Mol Biochem Parasitol.

[29] Gonen T, Walz T (2006). The structure of aquaporins.. Q Rev Biophys.

[30] Tsukaguchi H, Weremowicz S, Morton CC, Hediger MA (1999). Functional and molecular characterization of the human neutral solute channel aquaporin-9.. Am J Physiol.

[31] Ohgusu Y, Ohta KY, Ishii M, Katano T, Urano K (2008). Functional characterization of human aquaporin 9 as a facilitative glycerol carrier.. Drug Metab Pharmacokinet.

[32] Dimmer KS, Friedrich B, Lang F, Deitmer JW, Broer S (2000). The low-affinity monocarboxylate transporter MCT4 is adapted to the export of lactate in highly glycolytic cells.. Biochem J.

[33] Echevarria M, Windhager EE, Frindt G (1996). Selectivity of the renal collecting duct water channel aquaporin-3.. J Biol Chem.

[34] Ishibashi K, Kuwahara M, Gu Y, Kageyama Y, Tohsaka A (1997). Cloning and functional expression of a new water channel abundantly expressed in the testis permeable to water, glycerol, and urea.. J Biol Chem.

[35] Faghiri Z, Skelly PJ (2009). The role of tegumental aquaporin from the human parasitic worm, *Schistosoma mansoni*, in osmoregulation and drug uptake.. FASEB J.

[36] Katsura T, Verbavatz JM, Farinas J, Ma T, Ausiello DA (1995). Constitutive and regulated membrane expression of aquaporin 1 and aquaporin 2 water channels in stably transfected LLC-PK1 epithelial cells.. Proc Natl Acad Sci U S A.

[37] Preston GM, Carroll TP, Guggino WB, Agre P (1992). Appearance of water channels in *Xenopus* oocytes expressing red cell CHIP28 protein.. Science.

[38] Yukutake Y, Tsuji S, Hirano Y, Adachi T, Takahashi T (2008). Mercury chloride decreases the water permeability of aquaporin-4-reconstituted proteoliposomes.. Biol Cell.

[39] Tielens AG, van den Heuvel JM, van den Bergh SG (1990). Continuous synthesis of glycogen by individual worm pairs of *Schistosoma mansoni* inside the veins of the final host.. Mol Biochem Parasitol.

